# Global metabolic profile identifies choline kinase alpha as a key regulator of glutathione-dependent antioxidant cell defense in ovarian carcinoma

**DOI:** 10.18632/oncotarget.3589

**Published:** 2015-03-14

**Authors:** Anna Granata, Roberta Nicoletti, Paola Perego, Egidio Iorio, Balaji Krishnamachary, Fabio Benigni, Alessandro Ricci, Franca Podo, Zaver M. Bhujwalla, Silvana Canevari, Marina Bagnoli, Delia Mezzanzanica

**Affiliations:** ^1^ Unit of Molecular Therapies, Fondazione IRCCS Istituto Nazionale dei Tumori, Milan, Italy; ^2^ Molecular Pharmacology, Department of Experimental Oncology and Molecular Medicine, Fondazione IRCCS Istituto Nazionale dei Tumori, Milan, Italy; ^3^ Department of Cell Biology and Neurosciences, Istituto Superiore di Sanità, Rome, Italy; ^4^ Division of Cancer Imaging Research, In Vivo Cellular and Molecular Imaging Center, Russell H. Morgan Department of Radiology and Radiological Science, The Johns Hopkins University School of Medicine, Baltimore, MD, USA; ^5^ Division of Oncology, Urological Research Institute, IRCCS Ospedale San Raffaele, Milan, Italy

**Keywords:** Choline kinase, ovarian cancer, phosphocholine metabolism, glutathione, reversal of drug resistance

## Abstract

Epithelial Ovarian Cancer (EOC) “*cholinic phenotype”,* characterized by increased intracellular phosphocholine content sustained by over-expression/activity of choline kinase-alpha (ChoKα/CHKA), is a metabolic cellular reprogramming involved in chemoresistance with still unknown mechanisms.

By stable CHKA silencing and global metabolic profiling here we demonstrate that CHKA knockdown hampers growth capability of EOC cell lines both in vitro and in xenotransplant in vivo models. It also affected antioxidant cellular defenses, decreasing glutathione and cysteine content while increasing intracellular levels of reactive oxygen species, overall sensitizing EOC cells to current chemotherapeutic regimens. Natural recovering of ChoKα expression after its transient silencing rescued the wild-type phenotype, restoring intracellular glutathione content and drug resistance. Rescue and phenocopy of siCHKA-related effects were also obtained by artificial modulation of glutathione levels. The direct relationship among CHKA expression, glutathione intracellular content and drug sensitivity was overall demonstrated in six different EOC cell lines but notably, siCHKA did not affect growth capability, glutathione metabolism and/or drug sensitivity of non-tumoral immortalized ovarian cells. The “*cholinic phenotype”,* by recapitulating EOC addiction to glutathione content for the maintenance of the antioxidant defense, can be therefore considered a unique feature of cancer cells and a suitable target to improve chemotherapeutics efficacy.

## INTRODUCTION

Although the incidence is quite low, Epithelial Ovarian Cancer (EOC) is a highly lethal malignancy being the leading cause of gynecological cancer death [1]. Late diagnosis, frequent relapse and development of chemoresistance mainly contribute to the unfavourable prognosis [2]. As in many other cancer types, drug resistance in EOC is a complex phenomenon associated with several alterations that affect multiple factors and pathways [3, 4] including reprogram of cell metabolism to sustain cell growth and survival [5].

Aberrant metabolism has been recently proposed as a cancer hallmark [6], and it represents a source of promising therapeutic targets [7]. In particular, altered choline metabolism, characterized by increased phosphocholine (PCho) and total choline-containing compounds (tCho), is a metabolic hallmark that reflects the complex reciprocal interactions between oncogenic signaling and cellular metabolism [8, 9]. In recent years this altered choline metabolism, has been reported in different cancer types including breast, lung, prostate cancer [10-12] and more recently in pancreatic, endometrial cancer [13, 14] and also in glioma in association with R132H mutation of IDH1 gene [15]. These observations also provided the molecular basis for the development of non-invasive imaging approaches based on choline phosphorylation for the characterization of tumor growth and response to therapy [8, 16, 17] as well as the rationale for developing specific inhibitors for this metabolic pathways even in disease others than cancer [18, 19].

We described an increase in PCho levels also for EOC [20, 21] and demonstrated that intracellular PCho accumulation was sustained by activation of enzymes involved in phosphatidylcholine (PtdCho) biosynthetic and catabolic pathways. They include: choline kinase (ChoK), which produces PCho, and PtdCho-specific phospholipase C which, by degrading PtdCho, contributes to restore the PCho pool [20, 21]. Choline kinase alpha (ChoKα) plays a major role in increasing PCho content in EOC through increased activity and expression [20, 22], whereas the expression of the beta isoform of the enzyme (ChoKβ) remained unchanged [22]. We showed that transient down-modulation of ChoKα in EOC cells decreased cell growth, cell motility and invasion capability, overall weakening the EOC aggressive phenotype and also increasing sensitivity to cisplatin (cis-diammine-dichloro-platinum, DDP) and paclitaxel even in cells exhibiting intrinsic resistance to DDP like the SKOV3 cell line [23].

Among the various metabolism-related mechanisms affecting drug sensitivity, high intracellular levels of reduced glutathione (GSH) have been shown to contribute in developing resistance to chemotherapeutic drugs including DDP and doxorubicin [24-26]. GSH is a thiol peptide which tightly regulates cell redox status through its antioxidant and reducing activities [27]. Many conventional antitumor agents have been shown to trigger cancer cell death by inducing oxidative stress; therefore, targeting oxidative-stress related pathway is considered a promising tool to overcome drug resistance [27].

Our present analysis of the global metabolic profile of CHKA silenced cells disclosed altered glutathione metabolism. Here we demonstrate that ChoKα is directly involved in regulation of glutathione-dependent oxidative-stress signaling pathway in EOC cells and that targeting the ChoKα-mediated stress response increases the sensitivity of EOC cells to standard chemotherapeutics.

## RESULTS

### Stable CHKA silencing hampers *in vitro* and *in vivo* EOC aggressiveness and impairs PCho accumulation

**Figure 1:**
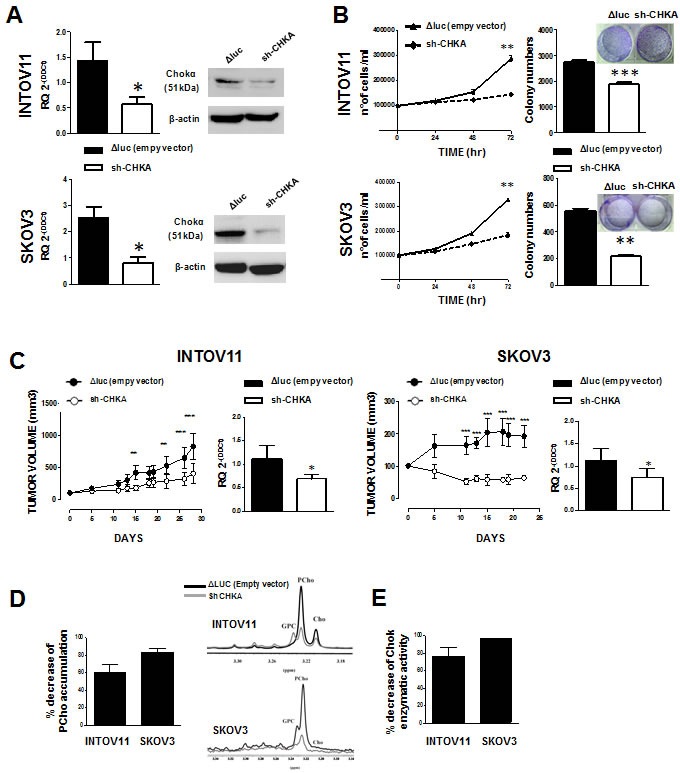
Functional and biological effects of CHKA stable silencing in EOC cell lines and in *in-vivo* tumor growth A: *Left panels*: expression of CHKA mRNA. qRT-PCR data (mean±SD) of 3 independent experiments for each cell line are reported (INTOV11 p=0.0193; SKOV3 p=0.019); GAPDH was used as housekeeping gene and I64-hTERT cell preparations as calibrator. *right panel*: representative immunoblot for Chokα protein expression. β-actin was used as loading control. B: Effects of sh-CHKA on *in vitro* cell growth. *Left panels*: proliferation curve; viable cells counted at 24, 48 and 72h post-seeding (INTOV11 p=0.0034; SKOV3 p=0.0036). *Right panels:* foci formation assay; quantification of the number of colonies after 10 days of growth is reported; in the inset is a representative image. Data are the mean ± SD of three independent experiments (INTOV11 p=0,0001; SKOV3 p=0.0038). C. *In vivo* tumor growth of transduced INTOV11 and SKOV3 cells. 3×10^6^ cells were injected sub-cutaneously into nude mice. *Left panels*: graphs are referred to volume in mm^3^ of tumors derived from ΔLuc (full circles, n=5) and sh-CHKA (open circles, n=5) transduced cells. *Right panels*: qRT-PCR for CHKA mRNA expression of tumors derived from INTOV11 (p=0.0244) and SKOV3 (p=0.0313) xenografts. Data are the mean ± SD of 5 tumors. D: Percentage of reduction of PCho accumulation quantify by analysis of 1H-MRS tCho profile in siCHKA INTOV11 and SKOV3 cells as compared to their relative controls. A representative 1H-MRS tCho profiles for each model is reported (GPC: glycerophosphocholine, PCho:phosphocholine, Cho: Choline). Black lines: ΔLuc-sh-RNA cells; grey lines: CHKA-sh-RNA cells. E. Percentage of reduction of Chok enzymatic activity in CHKA-sh-RNA INTOV11 and SKOV3 cells compared to their relative control.

To investigate the dynamics of long-term biological effects related to CHKA silencing, INTOV11 and SKOV3 cells were transduced with a lentiviral vector expressing GFP and specific CHKA shRNA [28]. A significant 61% ± 1% and 68.3% ± 7.6% reduction of CHKA mRNA was obtained in sh-CHKA transduced INTOV11 and SKOV3 cells respectively as compared to their relative control (ΔLuc) (Figure 1A left panels). A severe silencing effect was also observed at protein level where the densitometric analysis showed a proportional protein down-modulation of 40% ± 5% and 41% ± 9% on INTOV11 and SKOV3 transduced cell lines, respectively (Figure 1A, right panels). With the stable transfection approach we obtained a 44.4%±4.4% and 49.63%±1.76% *in vitro* growth inhibition (Figure 1B, left panels) and a 38%±10% and a 61.6%±6% reduction of colony formation (Figure 1B, right panels) in foci-formation assays for sh-CHKA INTOV11 and sh-CHKA SKOV3 respectively as compared to their relative controls. We observed in sh-CHKA transduced cells a 40% and 51% reduction of migration capability (Supplementary Figure 1A) and 41% and 45% inhibition of invasive potential (Supplementary Figure 1B) compared with their control cells, in INTOV11 and SKOV3 models, respectively. We also showed that stable CHKA silencing did not affect the main survival signaling pathways; indeed, phosphorylation level of the main molecules involved (Akt and ERK1/2 proteins) remained essentially unchanged in both sh-CHKA models as compared to their controls (Supplementary Figure 1C).

Given the strong inhibitory effects on *in*-*vitro* cell proliferation by CHKA stable silencing, we evaluated potential inhibitory effects in *in-vivo* models. Volumes of subcutaneously growing tumors were monitored and a significant inhibition of tumor growth was observed for both EOC silenced cell lines (Figure 1C, left panels). Tumors derived from control and sh-CHKA groups were then analyzed at molecular level. qRT-PCR analysis reported in Figure 1C (right panels) showed down modulation of CHKA mRNA expression in sh-CHKA xenografts of both INTOV11 and SKOV3 as compared to their relative controls.

CHKA-shRNA lentivirus transduction dramatically impacted on EOC choline metabolism. Fully relaxed ^1^H-MR spectra performed on water-soluble extracts, showed that PCho levels were significantly higher in ΔLuc-shRNA cells as compared to CHKA-shRNA transduced cells. Quantitative analysis showed a decrease of 61±9% and 83±3% of PCho content in sh-CHKA INTOV11 and SKOV3 cells respectively, as compared to their ΔLuc-shRNA controls (Figure 1D, left panel; representative examples are reported in middle and right panels). The indirect evidence of decreased ChoK-alpha activity (evaluated as decrease of PCho content) in sh-CHKA cells was confirmed by the direct measurement of enzymatic activity in both EOC models. Indeed, consistently with the reduction of PCho levels in sh-CHKA transduced cells a significant decrease of 77±16% and 97±32% of ChoK enzymatic activity as compaired to controls was detected in INTOV11 and SKOV3 cell lines (Figure 1E).

### CHKA silencing impairs EOC antioxidant cell defense

Global biochemical profiles, performed with the Metabolon technology platforms, were determined comparing ΔLuc and sh-CHKA cells for INTOV11 and SKOV3 EOC models collected 24 and 72 hours after plating. Principal component analysis of metabolic profile demonstrated distinct separation between the two cell lines and also clear clustering patterns associated with seeding times in both cell lines consistent with time-dependent metabolic adaptation (not shown). The greatest metabolic changes were observed at 72h post seeding where a number of metabolites were found to be down-regulated in CHKA silenced cells. A summary of biochemicals which achieved statistical significance (p≤0.05), as well as those approaching significance (0.05<p<0.10) is reported in Supplementary Figure 2A. The complete heatmap of significantly altered metabolites for each cell line is reported in Supplementary Figure 2B. We identified as commonly altered in the two sh-CHKA models, metabolic pathways mainly related to amino acid, lipid and nucleotide metabolism (see Supplementary Figure 2C for details).

**Figure 2:**
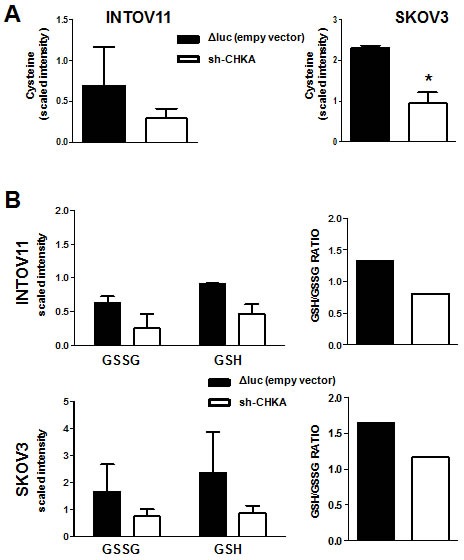
Stable CHKA silencing affected cysteine and glutathione intracellular content Quantification of metabolic signals in control (ΔLuc) and sh-CHKA EOC cell lines of: (A) Cysteine (SKOV3 p=0.0117); (B) GSSG and GSH. Data are mean ± SD of values obtained by mass spectrometry (Metabolon Technology) on 4 replicates analyzed 72h post seeding. GSH/GSSG ratio derived from this data is reported for each cell line in *right panels.*

**Figure 3:**
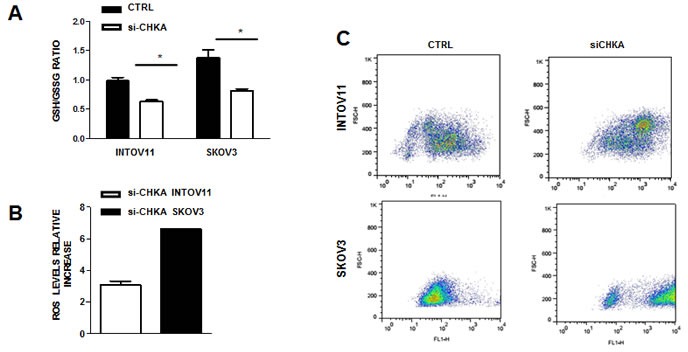
CHKA silencing regulates GSH content and ROS activation A: Assessment of GSH/GSSG ratio in transiently CHKA-silenced EOC cell lines using a luminescence-based assay B. Analysis of intracellular ROS levels. The relative increase in intracellular ROS levels in siCHKA silenced cells as compared to their controls is reported. ROS levels were measured by flow cytometry using the oxidation-sensitive probe CM-H2DCF-DA whose fluorescent signal intensity is directly proportional to production of reactive oxidants. To calculate the relative fold change of ROS signal in siCHKA vs control cells, unlabelled cells were used as negative control to derive the mean fluorescence index for each sample. C: A representative experiment for each cell line is reported showing the increase in fluorescence signaling (FL1-H) following siCHKA.

We found of particular interest the alterations related to glutathione and its precursor cysteine. Indeed, in this study CHKA stable silencing resulted in a significant decrease in cysteine levels as compared to the relative ΔLuc controls in both cellular models (Figure 2A), coupled with an alteration of glutathione metabolism, commonly characterized by a 2-fold decrease of oxidized glutathione (GSSG) content and a 3-fold decrease of reduced glutathione (GSH) (Figure 2B left panels). A 30-40% reduction of GSH/GSSG ratio indicated an overall decrease in GSH content in sh-CHKA cells as compared to their controls (Figure 2B, right panels). To independently validate this observation, we transiently silenced CHKA expression on INTOV11 and SKOV3 and quantified GSH and GSSG levels using a luminescence-based system assay, obtaining a 2 fold reduction of GSH/GSSG ratio in silenced (si-CHKA) cells compared to their relative controls (Figure 3A). GSH is known to regulate redox state through its antioxidant and reducing activities. Our observations suggested that CHKA knock down, by altering GSH intracellular levels, may cause an increase in ROS levels thus impairing antioxidant cellular defense and possibly enhancing cell responsiveness to drug treatment. We therefore assessed the ROS intracellular levels measuring by flow cytometry the fluorescent signal of CM-H2DCF-DA probe in control and si-CHKA cells. A 3-fold and a 6-fold increase in intracellular ROS levels was detected in INTOV11 and in SKOV3 siCHKA cells respectively as compared to their corresponding controls (Figure 3B and 3C).

**Figure 4:**
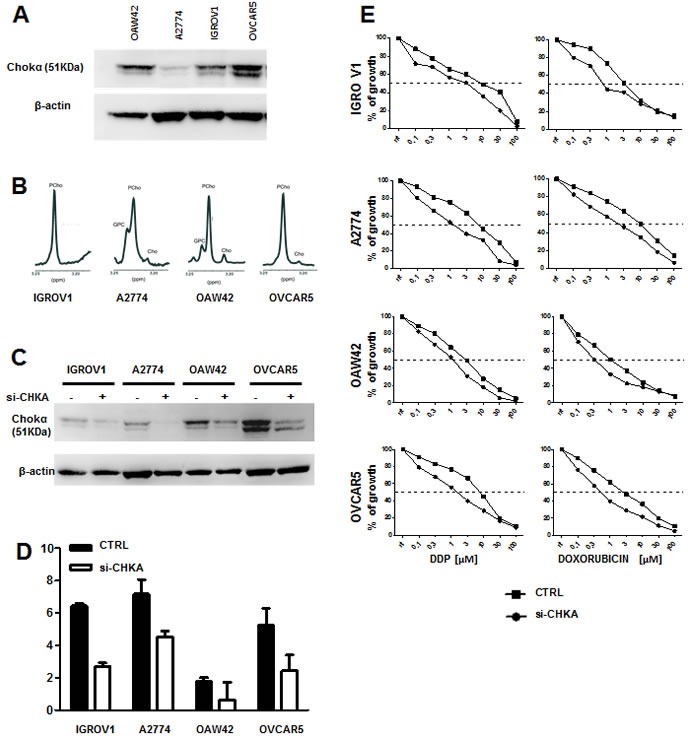
Alteration of GSH metabolism following CHKA silencing is a common feature of EOC cells Representative experiments in a panel of four EOC cell lines are shown: A. Immunoblot for CHKA protein expression. B. MRS spectra profile for PCho intracellular pooling. C. Assessment of Chokα downmodulation by Western blot analysis. D: Quantification of GSH and GSSG concentration by luminescence-based assay in control and siCHKA-EOC cell lines. Data are mean ± SD of 3 independent experiments. E. Sensitivity to DDP and Doxorubicin treatment were tested in a panel of EOC cell lines after CHKA transient silencing (siCHKA).

In order to exclude a cell line-dependent effect, CHKA was silenced in four different EOC cell lines: IGROV1, A2774, OAW42 and OVCAR5, which have heterogeneous ChoKα protein expression levels and PCho intracellular accumulation (Figure 4A and 4B).

In all tested cell lines, CHKA transient silencing induced an average 60% decrease of ChoKα protein expression (Figure 4C). Quantification of the GSH/GSSG ratio showed variable basal intracellular levels of these biochemicals, however a significant decrease of 2 fold of the GSH/GSSG ratio was confirmed after CHKA silencing in all models (Figure 4D). To exclude that these observations could be due to non specific off-target effects, decrease in GSH content was validated in siCHKA cells using a second independent *CHKA*-specific siRNA pool (Supplementary Figure 3).

To verify whether an altered expression of the cysteine transporter Sx_c_ could account for the observed decrease in cysteine, we analyzed the expression of the transporter subunit xCT/SLC7A11 both at mRNA and protein level (Supplementary Figure 4), and of the regulatory subunit SLC7A5 at mRNA level (not shown) and we did not observe any alteration following stable or transient CHKA silencing, thus excluding a decrease in cysteine uptake.

### CHKA silencing increases cell sensitivity to drugs by modulating intracellular GSH levels

CHKA silencing increased sensitivity to drug treatment in INTOV11 and SKOV3 [23] as well as in the four additional analyzed EOC cell lines (Figure 4E) where we validated the GSH decrease following CHKA down modulation (see Figure 4D).

**Figure 5:**
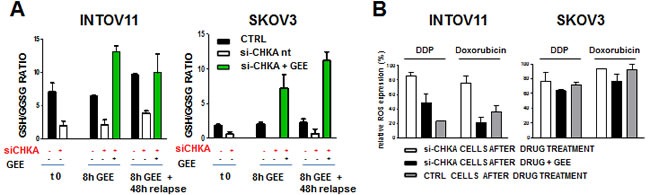

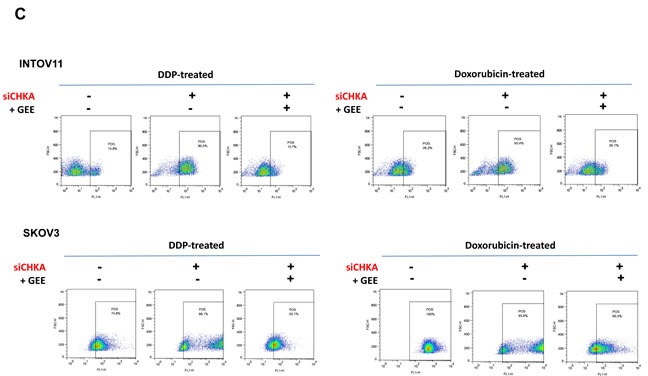

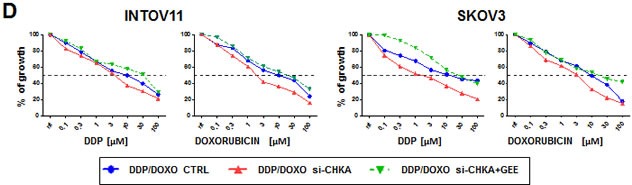
ROS levels and drug sensitivity are affected by modulations of intracellular GSH content mimicking CHKA silencing and/or expression A: Reloading of GSH content in siCHKA cells by 10 μM GEE treatment. Intracellular levels of reduced and oxidized glutathione (GSH/GSSG ratio) were monitored at the end of 72h of silencing (t0), after GEE loading (8h GEE) and at the end of treatment (8h GEE +48h relapse). B: GEE treatment decreases ROS levels in siCHKA- drug treated cells. Cells were treated with 10μM DDP or doxorubicin in presence or absence of GEE. Control (ctrl) drug-treated cells were used as negative control to measure the relative ROS level variation. ROS specific signal after GEE treatment of gated cells (see panel C) is indicated. C: A representative experiment is reported for both cell lines. For comparison with B: for each treatment left panels correspond to grey columns; middle panels to black columns and right panels to white columns. D: Effects of GEE exposure on cell sensitivity to DDP and doxorubicin in INTOV11 and SKOV3 CHKA silenced cells.

Since GSH intracellular levels are known to interfere with cell sensitivity to different antitumor agents including platinum compounds, we investigated whether the alteration of GSH/GSSG levels dependent upon CHKA silencing was the mechanism by which cell sensitivity was modulated. Thus, to phenocopy the effects of CHKA-silencing, we reduced basal intracellular glutathione levels treating wild type INTOV11 and SKOV3 cells with a non toxic concentration of BSO. As hypothesized, this GSH-depleting agent significantly increases DDP and doxorubicin sensitivity (Supplementary Figure 5 upper and lower panels, respectively), mimicking the pattern of sensitization observed after CHKA silencing (see Figure 4E and [23]). As complementary approach, we examined if we could rescue the resistant phenotype in INTOV11 and SKOV3 siCHKA cells by restoring the GSH intracellular levels. The glutathione tripeptide itself is not cell permeable, therefore to increase its intracellular content we loaded cells with a membrane/lipid permeable derivative, Glutathione Reduced Ethyl Ester (GEE) and we evaluated the capability of GEE treatment to recover intracellular GSH levels in siCHKA cells. After CHKA silencing, cells were loaded with GEE for 8h and then incubated for 48h in its absence, thus mimicking the time-schedule of the drug treatment. GSH/GSSG levels were monitored and quantified at the end of silencing (t0), after loading (8h GEE) and at relapse. CHKA silencing caused in both cell lines the expected two-fold decrease in the GSH/GSSG ratio as compared to relative controls (Figure 5A, t=0). GEE treatment effectively recovered GSH levels in both siCHKA cell lines and such an increase was maintained up to 48h after the end of GEE loading (Figure 5A, green histograms).

Most importantly GSH reloading in siCHKA cells substantially affected intracellular ROS levels, by lowering their amount to levels comparable to drug treated control cells (Figure 5B and 5C).

**Figure 6:**
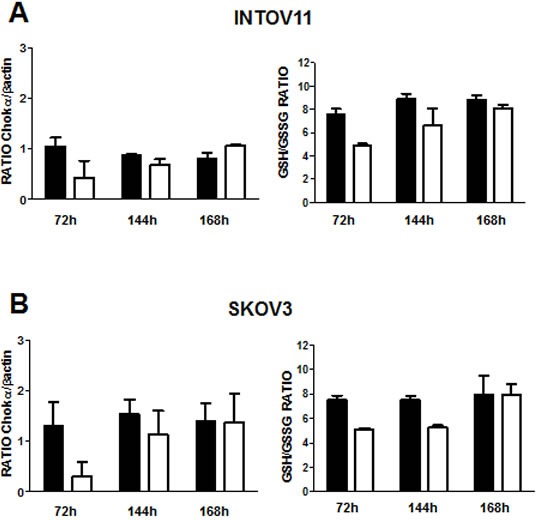

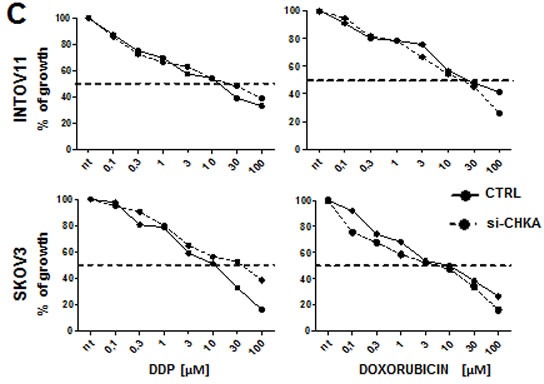
Recovering of ChoK expression rescues GSH content and cause loss of drug sensitization in siCHKA cells ChoKα protein expression (left panels) and GSH/GSSG ratio (right panels) were analyzed at 72, 144 and 168h after the end of CHKA transient silencing in INTOV11 (A) and SKOV3 (B) cells. Densitometric analysis of ChoKα content normalized to β-actin is reported. C. Sensitivity to DDP and doxorubicin treatment assessed at complete CHKA expression recovery (168h after the end of silencing).

Significantly, GEE treatment reverted drug sensitization associated with CHKA silencing, almost completely retrieving the dose-response curve typical of wt and control cells (Figure 5D). Rescue of GSH content and loss of drug sensitization were also observed after natural recovery of CHKA expression, 168h after the end of silencing (Figure 6A-6C).

### CHKA silencing does not affect Glutathione metabolism and/or drug sensitivity in non- tumoral ovarian cells

**Figure 7:**
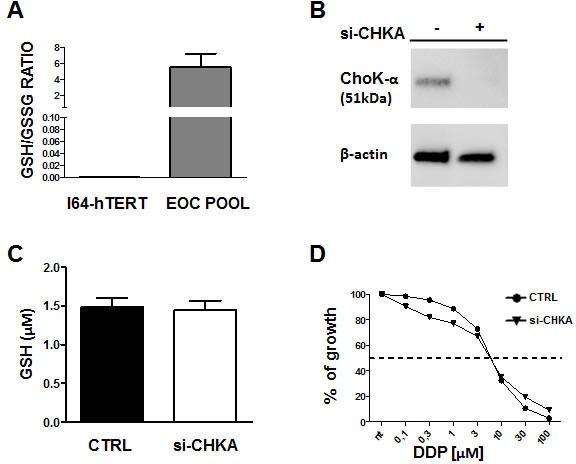
Alteration of Glutathione metabolism following CHKA silencing is a characteristic of tumoral cells A: Quantification of GSH/GSSG ratio in I64-hTERT non tumoral ovarian cell line and in a pool of EOC cells. B: Representative Western blotting analysis of ChoK-α expression on silenced I64-hTERT and control cells. β-actin is shown as a protein loading control. C: Quantification of GSH concentration, in I64-hTERT CHKA transiently silenced and control cells. D: Sensitivity to DDP in CHKA silenced I64-hTERT.

**Figure 8:**
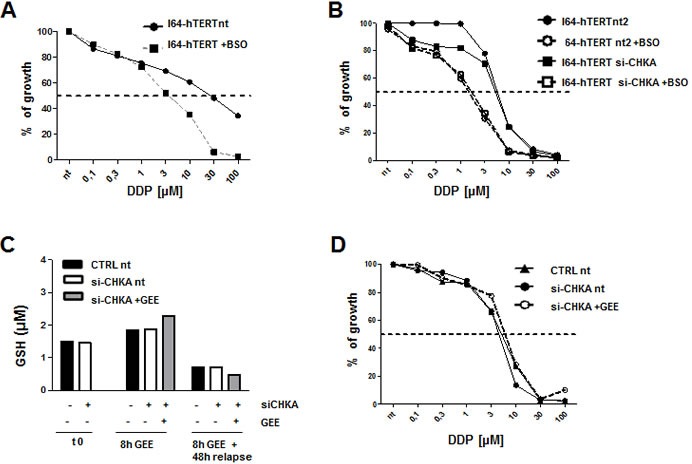
Effects of depletion and rescue of GSH in non tumoral I64-hTERT cells upon CHKA silencing A: Effects of BSO treatment on DDP sensitivity in I64-hTERT wt cells. Mean ± SD of growth percentage of treated vs. untreated cells is reported. B: Effects of BSO treatment on DDP sensitivity in I64-hTERT control and siCHKA cells. C: Quantification of GSH concentration at the end of 72h of silencing, after GEE loading and the end of drugs treatment. D: Effects of DDP treatment in presence or absence of GEE in siCHKA I64-hTERT cells. Mean ± SD of growth percentage of treated vs. untreated cells is reported.

To assess the specific biological impact of GSH regulation by CHKA expression in EOC cell lines, we evaluated the GSH/GSSG ratio in the non-tumoral ovarian cell line I64-hTERT and its possible alteration following CHKA silencing. The GSH/GSSG ratio was significantly higher in EOC cells than in I64-hTERT (Figure 7A) and knock down of CHKA in I64-hTERT cells decreased ChoK-alpha protein expression (Figure 7B) but it did not impact on GSH intracellular levels or drug sensitivity (Figure 7C and 7D). GSH depletion by BSO resulted in a sensitization of I64-hTERT cells to DDP, suggesting that in spite of its low basal level, intracellular GSH is involved in mediating drug cytotoxic effects. However, no differences were detected between CHKA silenced and non-silenced cells (Figure 8A and 8B). Indeed, GEE treatment caused a moderate increase in the GSH levels in I64-hTERT (Figure 8C), and accordingly no differences were observed in drug sensitivity of siCHKA, GEE-treated cells (Figure 8D).

## DISCUSSION

Aberrant choline metabolism is now a recognized metabolic hallmark for different cancer types [8]. We significantly contributed to define the relevance of this hallmark in EOC as well, showing that tumor cells have an increased expression and activation of ChoKα [20, 22] and that transient CHKA silencing affected cell behavior *in vitro*, hampering the aggressive phenotype [23]. These observations were overall suggestive for a primary role of altered Chok-α in sustaining the EOC “*cholinic phenotype”.*

Here we showed that a stable silencing of CHKA affected EOC *in vivo* growth, supporting the hypothesis that EOC aggressiveness may rely, at least in part, also on the expression and activity of this enzyme. We clearly showed that the depletion of CHKA expression was associated to a drop in PCho content and Chok activity, indicating a predominant role of the alpha isoform in the build-up of PCho in EOC cells. Furthermore, we did not observe any significant changes in the levels of PtdCho in CHKA-silenced cells (data not shown), suggesting that the phospholipid homeostasis was not substantially altered under these experimental conditions. ChoK-alpha activation has been described as a mediator of relevant survival signaling pathways (MAPK and AKT) and as a regulator of phosphatidic acid levels [29, 30]. We previously showed that in a transient siCHKA setting the phosphorylation status of the main signaling molecules was not affected by CHKA knock down. We now validated these observations in stable shCHKA cells where we also did not detect variation in phosphatidic acid content, further indicating that CHKA silencing affected different pathways in EOC cells.

The overall metabolic profiling disclosed an altered glutathione metabolism characterized by a decreased cysteine and GSH content in CHKA-silenced cells. Reduction of GSH content in CHKA silenced cells could be ascribed to limited cysteine availability or reduced activity/expression of the two enzymes which, starting from cysteine, are directly involved in glutathione synthesis: glutamate-cysteine ligase (GCL/GCS) and glutathione synthase (GS) [27]. Our MS data clearly showed a decreased cysteine content, thus supporting a primary role for this precursor in determining the GSH content. Limited cysteine availability could in turn be due to both impaired import by SXc carrier or impaired synthesis by the trans-sulphuration pathway. However upon CHKA knockdown we did not observed significant modification of the active transporter subunit of the SXc carrier (xCT) both at mRNA and protein level, thus suggesting that reduction of cysteine is not related to a decreased intake but might predominantly rely on decrease of the trans-sulphuration pathway. Furthermore, methionine could also participate (through the methionine cycle and folate metabolism) in the biosynthesis of the sulphur-containing amino acids homocysteine, cysteine, and taurine and then regulate the trans-sulphuration pathway and intracellular levels of glutathione [31]. Interestingly both methionine and folate metabolism pathway were found significantly modulated in the metabolic heatmap. Since the levels of methionine could influence proliferation as well as the drug resistance in cancer cells [32, 33], further studies are warranted to better clarify the link between the methionine and/or folate cycles and drug resistance in sh-CHKA cells.

The occurrence of drug resistance represents the major limit to the efficacy of chemotherapeutic treatment currently employed for progressive or recurrent EOC disease, such as a platinum-taxane (the standard first line therapy) or a platinum-liposomal doxorubicin combination [34]. However, the mechanisms of cytotoxic drug resistance in EOC remain elusive. Interestingly, a cytoprotective role has been proposed for high intracellular GSH content [24-26] and consistently, increased GSH content has been observed in different tumor types [27]. Indeed, besides participating in efflux transporter-mediated detoxification [26], GSH acts as scavenger of free radicals and peroxides accumulating in cell during oxidative stress like the hypoxic condition in tumor tissues. Therefore, treatments causing GSH depletion, by perturbing cell redox homeostasis, are expected to render tumor cells more susceptible to chemotherapeutic agents. In accordance with this proposed mechanism we observed that CHKA targeting increased intracellular ROS levels and sensitivity to DDP and doxorubicin treatment and these effects were mediated by decreased GSH content. Indeed, drug sensitivity acquired following CHKA silencing was lost by restoring, in si-CHKA cells, the GSH content both artificially or after natural recovery of Chok-α expression thus confirming the direct relationship existing among Chok-α expression, GSH content and drug sensitivity.

Malignant transformation is characterized by the progressive acquisition of genetic and epigenetic alterations, frequently involving hyperactivation of oncogenes and/or inactivation of oncosuppressor genes, which induce addiction to specific signaling and metabolic pathways [6]. Moreover, the overall deregulation of cellular processes and functions is frequently associated with enhanced cellular stress; thus malignant cells have to adapt to this phenotype, becoming dependent on a number of non-oncogenic functions to survive [7]. Several metabolic alterations of cancer cells such as their dependence on glutamine, glycine or serine might be considered as non-oncogenic addiction. Similarly, a dependency associated with ROS homeostasis has been shown to constitute a selective liability of malignant cells (as opposed to non-transformed cells) also in xenograft tumor models [35]. Identifying such dependencies represent a promising alternative for the development of new therapeutic strategies that by successfully targeting metabolic enzymes minimize adverse effects on normal tissues. Interestingly, the effects here described were observed in cancer cells but not in normal immortalized IOSE-hTERT cells, indicating that cancer cells only are dependent on Chok-α expression to maintain the ROS homeostasis. Indeed, we previously showed increased expression and activity of Chok-α in EOC cell lines and tumors as compared to the normal counterpart [20, 22] and glutathione levels have been reported to be higher in ovarian tumors than in normal ovarian tissue [36, 37]. Therefore CHKA targeting is expected to selectively affect EOC cells while sparing normal cells. The enhancement of cisplatin sensitivity upon CHKA knockdown in tumor cells might open promising clinical perspectives providing a chance for increasing the treatment efficacy by allowing reduction of the dose of administered drugs and limiting unwanted effects on normal cells.

Along this line, emerging technologies such as the use of biocompatible nanoparticles for systemic therapeutic gene silencing using RNA interference, offer new opportunities for targeting even those molecules otherwise undruggable.

## MATERIALS AND METHODS

### Cell lines

The following cell lines were used: INTOV11, obtained in our laboratory from a serous high-grade EOC [23], SKOV3, IGROV1, OVCAR5 (serous high-grade EOC) and A2774 (endometrioid histotype) obtained from ATCC; I64-hTERT normal ovary cell (IOSE-h-TERT) lines immortalized with hTERT obtained in our laboratory as described [20, 23]. For their relative maintenance and genotypic characterization see Supplementary Materials.

### Stable CHKA silencing

Strategy for cloning, generation and validation of stable cell lines expressing shRNA against CHKA and empty vector were as previously described [28]. Virions expressing CHKA-shRNA (sh-CHKA) and an empty vector control- ΔLuc-shRNA (Δluc) were used to transduce SKOV3 and INTOV11 cells.

### Transient CHKA silencing

CHKA silencing was performed using a specific siGENOME Smart Pool small interfering RNA (siRNA) targeting a sequence of CHKA gene different from the one recognized by the shRNA. A non targeting siRNA was used as control (Thermo Scientific, Dharmacon Inc, Chicago, IL, USA). Transfection was carried out as described [23]. Specificity of silencing effects was verified using the QIAGEN siRNA pool targeting *CHKA*. Details are reported in Supplementary Materials.

### Nuclear Magnetic Resonance (NMR) spectroscopy

Analyses were performed as described [20, 22] and a brief description is reported in Supplementary Materials.

### Enzymatic ChoK activity

^1^H and ^31^P NMR assays were performed upon addition of exogenous choline chloride, ATP and Mg^++^ ions to cytosolic cell preparations in Tris-HCl, pH 8.0, as described [20, 38].

### Western blotting; qRT-PCR; cell migration and cell invasion assays; drug sensitivity assays

For these assays, methods were essentially as described [23] and are described in details in Supplementary Materials with the list of specific antibodies, probes and reagents used.

### Growth and colony formation assay

Cells (100.000/well) were seeded in triplicates on 12-well plates. After transfection cell growth was assessed at each time-point by Trypan Blue exclusion assay using Countess automated cell counter (Invitrogen). Cell viability was evaluated as percentage of live cells in the total cell population. For colony formation assay, 5.000 cells were plated in 6 well plates and grown 10 days. Colonies were fixed with 10% (v/v) methanol for 10min at −20°C, stained with 0.5% Crystal violet solution (made in 25% methanol) for 10min at room temperature and washed with ddH_2_O for colony visualisation. Colonies were quantified using Odyssey Infrared laser scanner (Li-Cor Biosciences, NE, USA) at Infrared wavelength of 700 nm.

### Xenograft models

For both INTOV11 and SKOV3 cellular models, 3×10^6^ ΔLuc-sh-RNA and CHKA-sh-RNA -transduced cells were injected sub-cutaneously into 8 weeks-old female CD1 *Nu/Nu* mice (Charles River Laboratories) acclimatized for at least one week before tumor cell injection. Five animals/groups were used and monitored for up to 30 days from injection. Tumors were measured with a caliper and volume was calculated according to the following formula: volume (mm^3^)^=^ $\left(\frac{4\pi }{3}\right)\;\frac{d^{2}}{2}\;\frac{D}{2}$ .(D= major nodule diameter; d= minor nodule diameter). The mice at the end of the xenograft growth experiments were sacrified upon induction of surgical anesthesia with ketamine/xylazine. All protocols were approved by the Ethics Committee for Animal Experimentation of the Fondazione IRCCS Istituto Nazionale dei Tumori and performed according to institutional and the new Guidelines for the Welfare and Use of Animals in Cancer Research (D.L. 116/92 and subsequent implementing circulars).

### Metabolomic profiling

Metabolomic profiling analysis was performed by Metabolon as previously described [39] analyzing 4 replicates of parental, ΔLuc-sh-RNA and sh-CHKA transduced INTOV11 and SKOV3 cells collected 24 and 72 hours post seeding. Extracts from all samples were analyzed on the gas chromatography/mass spectrometry (GC/MS) and Liquid chromatography/mass spectrometry (LC/MS/MS) platforms. Details on: sample accessioning and preparation, GC/MS and LC/MS/MS platforms, quality assurance data extraction and compound identification are in Supplementary Material. The present dataset comprises a total of 207 compounds of known identity (biochemicals). Metabolon determined global biochemical profiles of the samples and compared them across treatment groups and cell lines to identify biochemicals which differed significantly between experimental groups within each cell line. Pathways were assigned for each metabolite, allowing examination of overrepresented pathways. All statistical analyses were performed by Metabolon.

### GSH/GSSG quantification

After 72 hours of CHKA transfection, 1×10^4 cell were seeded in 96 well plate in FCS-free culture media and after attachment, cells were lysed and processed for the assay. Reduced and oxidized glutathione ratio was measured using the luminescent GSH/GSSG-Glo assay kit (Promega) according to the manufacturer's protocol. Luminescence was measured using an Ultra multiplate reader (Tecan Group, Mannedorf/Zurich, Switzerland). GSH/GSSG ratios are calculated from RLU measurements after interpolation of glutathione concentrations from standard curves.

### Glutathione depletion and loading

Buthionine sulfoximine (BSO; Sigma Aldrich; St Louis, MO, USA), an inhibitor of gamma-glutamylcysteine synthetase [40], was used for GSH depletion. 7,5×10^3^ cells/well were seeded in 96 well plates pre-incubated with or without 100 μM of BSO for 24 hours in FCS-free medium and then treated with cisplatin or doxorubicin and cytotoxicity was evaluated after 48h from medium replacement as above described. Glutathione mono-ethyl ester (L-gamma-glutamyl-L-cysteinylglycyl ethyl ester) (GEE; Sigma Aldrich; St Louis, MO, USA), a membrane/lipid permeable derivative of GSH [41, 42], was used for glutathione reloading experiments. To verify the efficacy of GSH reloading, 1×10^4^ cells/well were seeded in 96 well plate, pre-incubated with or without 10 μM of GEE for 8 hours and then processed for GSH/GSSG quantification according to kit's instructions. To verify the effects on drug sensitivity, 7,5×10^3^ cells/well were seeded in 96 well plates and pre-incubated with or without 10 μM of GEE for 1 hour followed by exposure to drugs for 7 hours; sensitivity to drugs was evaluated 48 hours later as above described.

### ROS detection

Intracellular accumulation of ROS was determined under basal condition and after specific treatments (CHKA silencing, drug treatment, GSH depletion/restoration) using the oxidation sensitive fluorescent probe 5-(and-6)-chloromethyl-2¢,7¢-dichlorodihydrofluorescein diacetate, acetyl ester (CM-H2DCF-DA) (Molecular Probes, Life Technologies Milan, Italy). Cells were harvested by trypsinization, washed and incubated for 45 min with 5 mM CMH2DCFDA and immediately analyzed by flow cytometry. As positive control we used cells pretreated with 150 μM H_2_O_2_ for 2hours and subsequently probed with CMH2DCFDA. Samples were acquired with FACS Canto (Becton - Dickinson) and the data processed using Flow Jo 7.6.4 Analysis Software (Becton-Dickinson).

### Statistical analysis

Data were analyzed using GraphPad Prism Software Version 5 (GraphPad Software, Inc, San Diego). Statistical significance was determined by one-way ANOVA or by Student's t-test, as specified. Differences were considered significant at P < 0.05. Asterisks in all denote a statistically significant difference in comparison to the corresponding control * *P* < 0.05; ** *P* < 0.01; *** *P* < 0.001. Data reported are the mean ±SD of at least three independent experiments unless otherwise indicated.

## SUPPLEMENTARY MATERIAL AND FIGURES


