# Supplementary material for: Global metabolic profile identifies choline kinase alpha as a key regulator of glutathione-dependent antioxidant cell defense in ovarian carcinoma
Source: Oncotarget. 2015 Mar 14;6(13):11216–30. doi: 10.18632/oncotarget.3589 (PMC4484451; doi:10.18632/oncotarget.3589)
Supplement: Supplementary file 1 [file oncotarget-06-11216-s001.pdf]

Global metabolic profile identifies choline kinase alpha as a key regulator of glutathione-dependent antioxidant cell defense in ovarian carcinoma

Supplementary Material

Cell lines

INTOV11, SKOV3, IGROV1, OVCAR5 cell lines were maintained in RPMI 1640 (Sigma, St. Louis, MO) supplemented with 10% (v/v) FCS and 2 mmol/L L-glutamine (Sigma, St. Louis, MO). OAW42 cells (serous histotype, kindly provided by Dr A. Ullrich, Max Planck Institute of Biochemistry, Martinsried, Germany) were cultured in MEM (Sigma Aldrich) supplemented with 10% FCS, 2 mmol l-glutamine and non essential amino acids. I64-hTERT normal ovary cell (IOSE-h-TERT) lines immortalized with hTERT, was maintained in 199-MCDB105 medium (Sigma, St. Louis, MO) supplemented with 10% FCS, 2 mmol l-glutamine, 200 µg/ml G418. Cells were cultured at 37°C in a humidified incubator under 5% CO_2 and genotyped at Functional Genomic facility of our Institute, using Stem Elite ID System (Promega), according to manufactures instructions and ATCC guidelines. Cells were routinely confirmed to be mycoplasma-free by Hoechst staining and MycoAlert Mycoplasma Detection Kit (Lonza).

Transient CHKA silencing

Cells (180.000/well) were seeded on 6-well plate and transfected with a final concentration of 40 nM specific small interfering RNA (siRNA) against CHKA, NM_212469 and NM_001277 (siGENOME Smart Pool, Thermo Scientific, Dharmacon Inc, Chicago, IL, USA), whereas a non-targeting siRNA was used as control (siGENOME Non-targeting siRNA#2, Dharmacon). Transfection was carried out using Lipofectamine2000 (Invitrogen), as described (paper ref. 13). The efficacies of CHKA silencing as well as its related biological effects were assessed at day 3 after 72h transfection without removing the transfection mixture. Specificity of silencing and absence of off-target effects were verified using the FlexiTube Gene Solution (QIAGEN) siRNA

pool targeting *CHKA* (NM_001277), together with their relative negative controls siRNA (QIAGEN), applying the same transfection protocols and siRNA concentration.

NMR spectroscopy.

Cells grown to 60-70% confluence were trypsinized 24h after culture medium change, counted, and assessed for cell viability (80-90%) and membrane integrity by trypan blue staining. Cell aqueous extracts derived from 5-10 x 10^6 cells/sample were prepared in EtOH 70% according to an established protocol (paper ref.12). Briefly, after ultra-sonication at 20 kHz, supernatants were lyophilized twice and the residue resuspended in 0.7 ml D_2O (Sigma-Aldrich, St. Louis, MO, USA) containing 0.1 mM 3-(trimethylsilyl)-propionic-2,2,3,3-d4 acid sodium salt as internal chemical shift and peak area standard. High-resolution NMR experiments (25°C) were performed at 9.4T (Bruker AVANCE spectrometer, Karlsruhe, Germany). ^{1}H-NMR spectra of cell extracts were acquired using 90° flip angle, 30 s repetition time, 32K time domain data points and 128 transient (paper ref.10).

Western blotting

Western blot analysis was performed as previously described (paper ref.13). Briefly, blots were probed with rabbit polyclonal antibodies anti-ChoKα (Sigma Aldrich; St Louis, MO, USA) at 1:300 dilution and anti-xCT (Abcam; ab37185) at 1:1000 dilution, both in 5% nonfat dry milk overnight at 4°C. Rabbit polyclonal anti β-actin antibody (Sigma Aldrich; St Louis, MO, USA) at 1:400 was used as loading control. The following primary antibodies were used at the manufacturing recommended concentrations: anti-MAP kinase activated, anti-phosphoAkt (Ser473) clone D9E, anti-Akt, (Cell Signaling Technology; New England BioLabs, Beverly, MA); anti-MAP kinase (ERK1/2), (Santa Cruz Biotecnology; Santa Cruz, CA). Appropriate horseradish peroxidase (HRP) conjugated secondary antibody were used at 1:2500 dilution in milk. Blots were acquired with a Bio-Rad apparatus using ChemiDoc XRS (Bio-Rad Laboratories Srl) and analyzed using Quantity One software (Biorad, Hercules, CA, USA). For densitometric analysis, protein levels were normalized to β-actin.

Quantitative Real Time PCR (qRT-PCR)

Total RNA was extracted from EOC cell lines using the RNA Spin Mini Isolation Kit (GE Healthcare Europe GmbH) following the manufacturer's protocol. cDNA was generated from 2µg of RNA using High-Capacity cDNA Archive Kit (Applied Biosystems) and qRT-PCR was carried out in triplicate using the 7900 HT FAST Real-Time PCR System (Applied Biosystems). Probes used for amplification were from Applied Biosystems (CHKA HS00608045_m1; xCT/SLC7A11 HS00204928_m1 and GAPDH HS03929097_g1). The ΔΔCT method was used to determine the quantity of the target sequences. In all qRT-PCR experiments *GAPDH* was amplified as endogenous control and I64-hTERT cells were used as calibrator. Analyses were performed using SDS software 2.2.2 (Applied Biosystems).

Drug treatment assays

For cytotoxicity assays, stably silenced or transient silenced cells 72 hours after transfection, were seeded at $7.5x10^4$ cells/well in 96-well flat-bottom plates and exposed for 7h to cisplatin (DDP) (TEVA Italia s.r.l) at serial dilution from $1x10^{-4}$ to $1x10^{-7}$ M, or doxorubicin (doxorubicin hydrochloride Pfizer, Italia s.r.l) at serial dilution from $1x10^{-4}$ to $1x10^{-7}$ M. Effects of drugs treatment were assessed 48h later using a CellTiter-GLO luminescent cell viability assay performed according to the manufacturer's instructions (Promega, Madison, WI). Luminescence was measured using an Ultra multiplate reader (Tecan Group, Mannedorf/Zurich, Switzerland).

Cell migration assays

Migration assays were performed using 24-Well Fluorimetric Cell Migration Assay (Merck Millipore, MA, USA). Cells were seeded in the upper chamber at 5×10^5 cells/ml in 0.3 ml serum-free culture media. Media supplemented with 10% FCS was placed as a chemoattractant in the bottom well in a volume of 0.5 ml. After incubation for 24 h at 37°C in an atmosphere containing 5% CO2, assay was performed according to the manufacturer's instructions (Merck Millipore, MA, USA). Migration was measured using a microplate reader (Tecan Group, Mannedorf/Zurich, Switzerland) with excitation/emission wavelengths of 485/535nm.

Cell invasion assays

Invasion capability was assayed using Fluoro Blok system in a 24-well companion plate (from Becton Dickinson) coated with Matrigel. 5×10^4 cells/well were seeded onto the apical surface of the inserts in 0.75 ml of serum-free colture media. The basal chamber of the wells was filled with 650 μL of media supplemented with FCS as chemoattractant. Cells were incubated in the Fluoro-Blok multiwell insert system for 24 hours at 37°C in a humidified atmosphere of 5% CO_2 and labeled with 4g/ml calcein AM (Molecular Probes, Life Technologies) in Hanks' balanced salt solution (Life Technologies) for 1 hour at 37°C and 5% CO_2. Fluorescence was measured using a microplate reader (Tecan Group, Mannedorf/Zurich, Switzerland) with excitation/emission wavelengths of 485/535nm.

Metabolomic profiling

Metabolomic profiling analysis was performed by Metabolon as previously described (17) analyzing 8 replicates of parental, ΔLuc-sh-RNA and sh-CHKA transduced INTOV11 and SKOV3 cells collected 24 and 72 hours post seeding. Extracts from all samples were split into equal parts for analysis on the gas chromatography/mass spectrometry (GC/MS) and Liquid chromatography/mass spectrometry (LC/MS/MS) platforms. *Sample Accessioning*. Each sample received was accessioned into the Metabolon LIMS system and was assigned by the LIMS a unique identifier that was associated with the original source identifier only. This identifier was used to track all sample handling, tasks, results etc. The samples (and all derived aliquots) were tracked by the LIMS system. All portions of any sample were automatically assigned their own unique identifiers by the LIMS when a new task is created; the relationship of these samples is also tracked. All samples were maintained at -80 ºC until processed. *Sample Preparation*. Samples were prepared using the automated MicroLab STAR® system from Hamilton Company. A recovery standard was added prior to the first step in the extraction process for QC purposes. Sample preparation was conducted using aqueous methanol extraction process to remove the protein fraction while allowing maximum recovery of small molecules. The resulting extract was divided

into four fractions: one for analysis by UPLC/MS/MS (positive mode), one for UPLC/MS/MS (negative mode), one for GC/MS, and one for backup. Samples were placed briefly on a TurboVap® (Zymark) to remove the organic solvent. Each sample was then frozen and dried under vacuum. Samples were then prepared for the appropriate instrument, either UPLC/MS/MS or GC/MS. *Ultrahigh performance liquid chromatography/Mass Spectroscopy (UPLC/MS/MS).* The LC/MS portion of the platform was based on a Waters ACQUITY ultra-performance liquid chromatography (UPLC) and a Thermo-Finnigan linear trap quadrupole (LTQ) mass spectrometer, which consisted of an electrospray ionization (ESI) source and linear ion-trap (LIT) mass analyzer. The sample extract was dried then reconstituted in acidic or basic LC-compatible solvents, each of which contained 8 or more injection standards at fixed concentrations to ensure injection and chromatographic consistency. One aliquot was analyzed using acidic positive ion optimized conditions and the other using basic negative ion optimized conditions in two independent injections using separate dedicated columns. Extracts reconstituted in acidic conditions were gradient eluted using water and methanol containing 0.1% formic acid, while the basic extracts, which also used water/methanol, contained 6.5mM Ammonium Bicarbonate. The MS analysis alternated between MS and data-dependent MS^2 scans using dynamic exclusion. Raw data files are archived and extracted as described below. *Gas chromatography/Mass Spectroscopy (GC/MS).* The samples destined for GC/MS analysis were re-dried under vacuum desiccation for a minimum of 24 hours prior to being derivatized under dried nitrogen using bistrimethyl-silyl-triflouroacetamide (BSTFA). The GC column was 5% phenyl and the temperature ramp was from 40° to 300° C in a 16 minute period. Samples were analyzed on a Thermo-Finnigan Trace DSQ fast-scanning single-quadrupole mass spectrometer using electron impact ionization. The instrument was tuned and calibrated for mass resolution and mass accuracy on a daily basis. The information output from the raw data files was automatically extracted as discussed below.

*Quality assurance/QC***.** For QA/QC purposes, additional samples were included with each day's analysis. These samples included extracts of a pool of well-characterized human plasma, extracts of

a pool created from a small aliquot of the experimental samples, and process blanks. QC samples were spaced evenly among the injections and all experimental samples were randomly distributed throughout the run. A selection of QC compounds was added to every sample for chromatographic alignment, including those under test. These compounds were carefully chosen so as not to interfere with the measurement of the endogenous compounds. *Data extraction and compound identification*. Raw data was extracted, peak-identified and QC processed using Metabolon's hardware and software. These systems are built on a web-service platform utilizing Microsoft's .NET technologies, which run on high-performance application servers and fiber-channel storage arrays in clusters to provide active failover and load-balancing (3). Compounds were identified by comparison to library entries of purified standards or recurrent unknown entities. Metabolon maintains a library based on authenticated standards that contains the retention time/index (RI), mass to charge ratio (*m/z)*, and chromatographic data (including MS/MS spectral data) on all molecules present in the library. Furthermore, biochemical identifications are based on three criteria: retention index within a narrow RI window of the proposed identification, nominal mass match to the library ± 0.2 amu, and the MS/MS forward and reverse scores between the experimental data and authentic standards. The MS/MS scores are based on a comparison of the ions present in the experimental spectrum to the ions present in the library spectrum. While there may be similarities between these molecules based on one of these factors, the use of all three data points can be utilized to distinguish and differentiate biochemicals. More than 3500 commercially available purified standard compounds have been acquired and registered into LIMS for distribution to both the LC and GC platforms for determination of their analytical characteristics. *Statistical Analysis:* Missing values (if any) are assumed to be below the level of detection. However, biochemicals which were detected in all samples from one or more groups but not in samples from other groups were assumed to be near the lower limit of detection in the groups in which they were not detected. In this case, the lowest detected level of these biochemicals was imputed for samples in which that biochemical was not detected. Following log transformation and imputation with

minimum observed values for each compound, ANOVA contrasts were used to identify biochemicals that differed significantly between experimental groups.

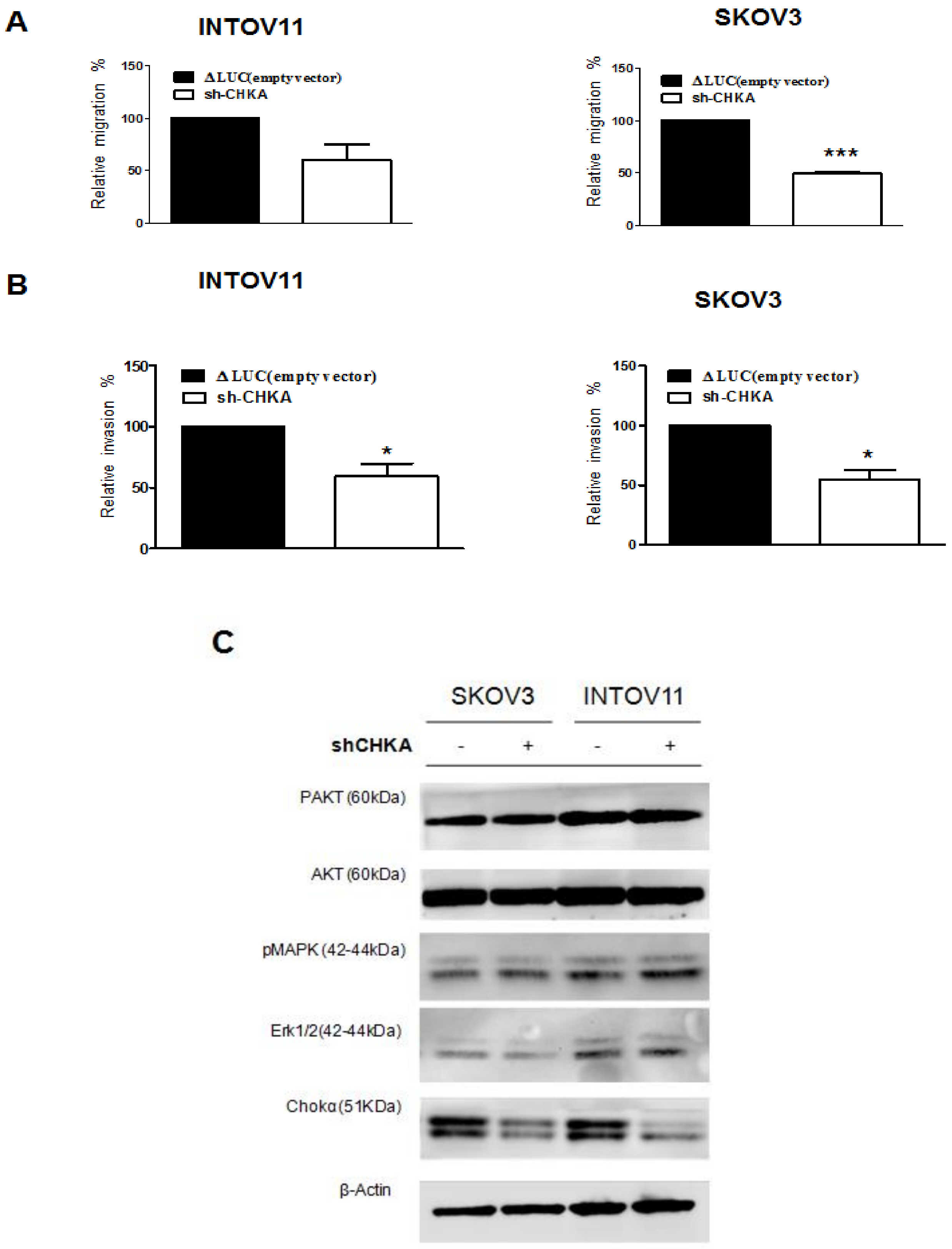

Supplementary Figure 1: Effects of CHKA stable silencing on migration and invasion capability

A: Migration assay. Cells migrating through the membrane were measured by relative fluorescence units (RFU) of CyQUANT dye binding by cellular nucleic acids in the lysates. The percentage of migration in sh-CHKA transduced INTOV11 and SKOV3 cells as compared to their relative controls (ΔLuc-sh-RNA) is reported (SKOV3 p=0.0006). **B:** Invasion assay. Cells invading through the membrane were measured by Odyssey Infrared laser scanner quantification after SRB staining. Invasion capability was calculated respect to control transduced (ΔLuc-sh-RNA) cells set to 100%. Mean relative percentage ± SD of 3 independent experiments is reported (INTOV11 p=0.0215; SKOV3 p=0.0267)**.** **C:** Effects of stable CHKA silencing on survival pathways activation. A representative western blot of Akt and ERK1/2 phosphorylation following CHKA knockdown in INTOV11 and SKOV3 cells is reported. β-actin is shown as a protein loading control.

A

Summary Statistics				
ANOVA Contrasts shRNA / Mock	Total biochemicals $p \leq 0.05$	Biochemicals (↑↓)	Total biochemicals $0.05 < p < 0.10$	Biochemicals (↑↓)
INTOV11	89	8 | 81	29	2 | 20
SKOV3	32	0 | 32	29	0 | 29

B

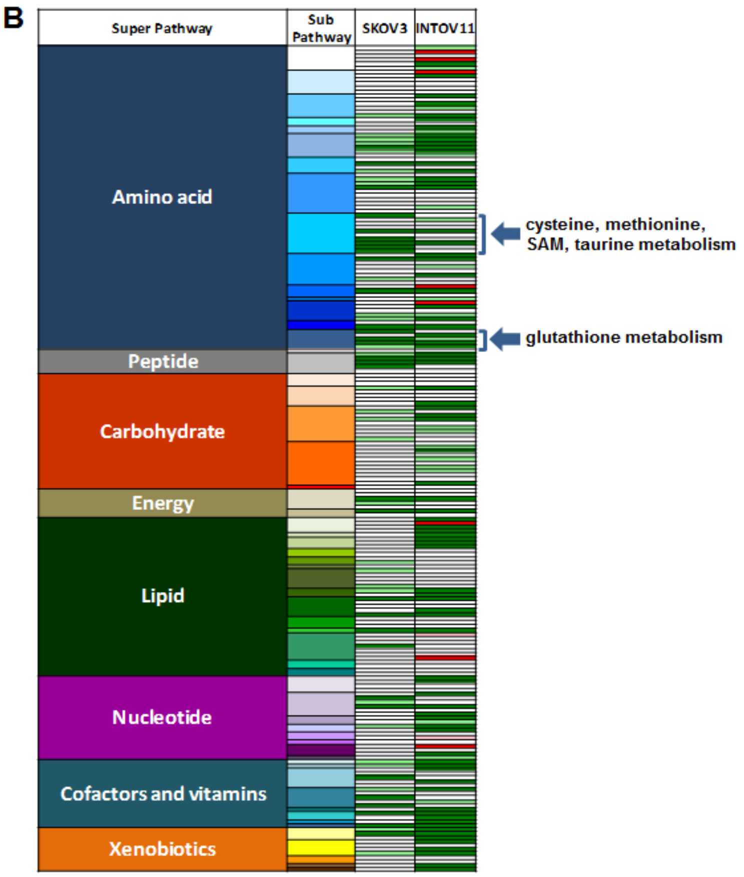

C

Super Pathway	Sub Pathway
Amino acid	Glycine, serine and threonine metabolism
	Alanine and aspartate metabolism
	Glutamate metabolism
	Histidine metabolism
	Lysine metabolism
	Phenylalanine & tyrosine metabolism
	Tryptophan metabolism
	Valine, leucine and isoleucine metabolism
	Cysteine, methionine, SAM, taurine metabolism
	Urea cycle; arginine-, proline-, metabolism
	Creatine metabolism
	Butanoate metabolism
	Polyamine metabolism
	Guanidino and acetamido metabolism
	Glutathione metabolism
Peptide	Dipeptide
	gamma-glutamyl
Carbohydrate	Aminosugars metabolism
	Fructose, mannose, galactose, starch, and sucrose metabolism
	Glycolysis, gluconeogenesis, pyruvate metabolism
	Nucleotide sugars, pentose metabolism
	Nucleotide sugars
Energy	Krebs cycle
	Oxidative phosphorylation
Lipid	Essential fatty acid
	Medium chain fatty acid
	Long chain fatty acid
	Fatty acid, dicarboxylate
	Fatty acid metabolism (also BCAA metabolism)
	Fatty acid metabolism
	Carnitine metabolism
	Bile acid metabolism
	Glycerolipid metabolism
	Inositol metabolism
	Ketone bodies
	Lysolipid
	Sphingolipid
	Sterol/Steroid

Super Pathway	Sub Pathway
Nucleotide	Purine metabolism, (hypo)xanthine/inosine containing
	Purine metabolism, adenine containing
	Purine metabolism, guanine containing
	Purine metabolism, urate metabolism
	Pyrimidine metabolism, cytidine containing
	Pyrimidine metabolism, orotate containing
	Pyrimidine metabolism, uracil containing
	Purine and pyrimidine metabolism
Cofactors and vitamins	Biotin metabolism
	Folate metabolism
	Nicotinate and nicotinamide metabolism
	Pantothenate and CoA metabolism
	Pyridoxal metabolism
	Riboflavin metabolism
	Thiamine metabolism
	Vitamin B6 metabolism
Xenobiotics	Benzoate metabolism
	Chemical
	Drug
	Food component/Plant
	Sugar, sugar substitute, starch

Supplementary Figure 2: Summary of significantly altered metabolites and biochemical pathways.

A Biochemicals that differed significantly between experimental groups (control Δluc and stably silenced sh-CHKA) within each cell line at 72h after plating. A summary of the numbers of biochemicals that achieved statistical significance ($p \leq 0.05$), as well as those approaching significance ($0.05 < p < 0.10$), is shown; downregulated metabolites are in green, upregulated are in red. **B** Heatmap of the metabolites significantly altered in sh-CHKA INTOV11 and SKOV3 cell lines as compared to their controls. Downregulated biochemicals with statistical significance $p \leq 0.05$ are in dark green, those approaching significance ($0.05 < p < 0.10$) are reported in light green. The same criteria are applied for up-regulated biochemicals, which are respectively in dark and light red. Major metabolic pathways (Super pathway) are indicated and detailed description of their specific sub-pathways is reported in panel **C.**

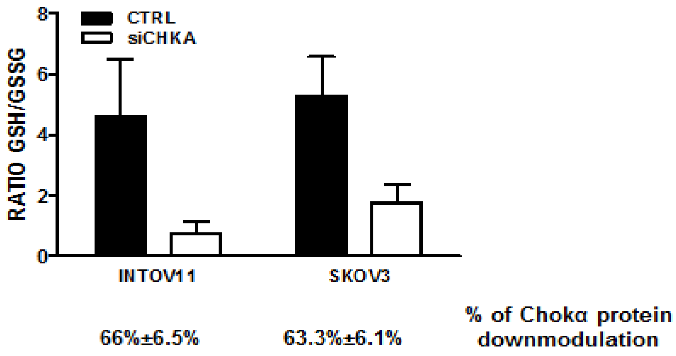

Supplementary Figure 3: Modulation of GSH/GSSG ratio using an independent CHKA siRNA pool.

GSH/GSSG ratio decrease was observed upon siCHKA using an alternative CHKA-specific siRNA pool. Percentage of Chokα protein decrease is reported below histograms.

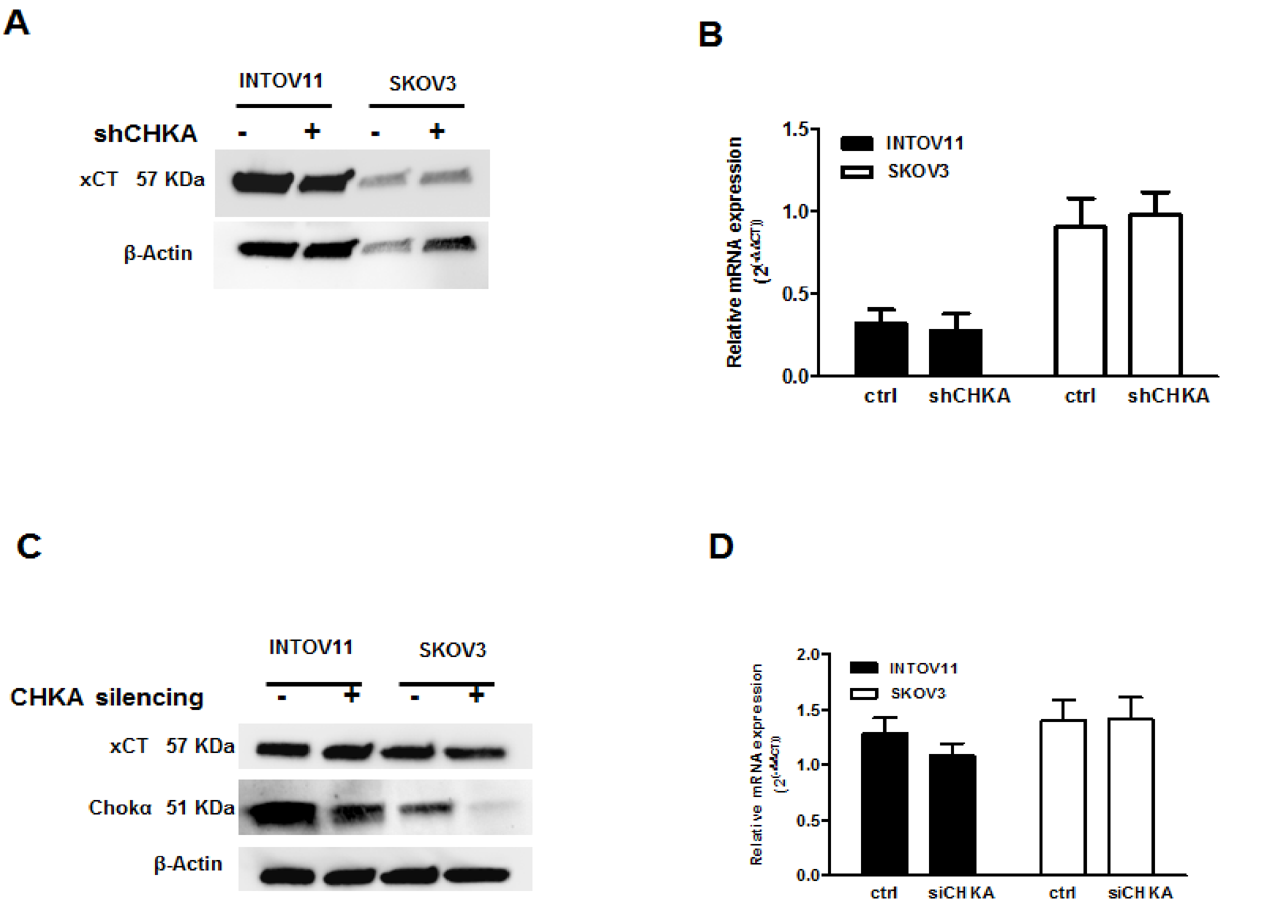

Supplementary Figure 4. Analysis of xCT expression.

(A-C) Western Blot and **(B-D)** mRNA analysis of xCT expression in INTOV11and SKOV3 cell lines stably (A,B) or transiently (C,D) silenced for CHKA expression.

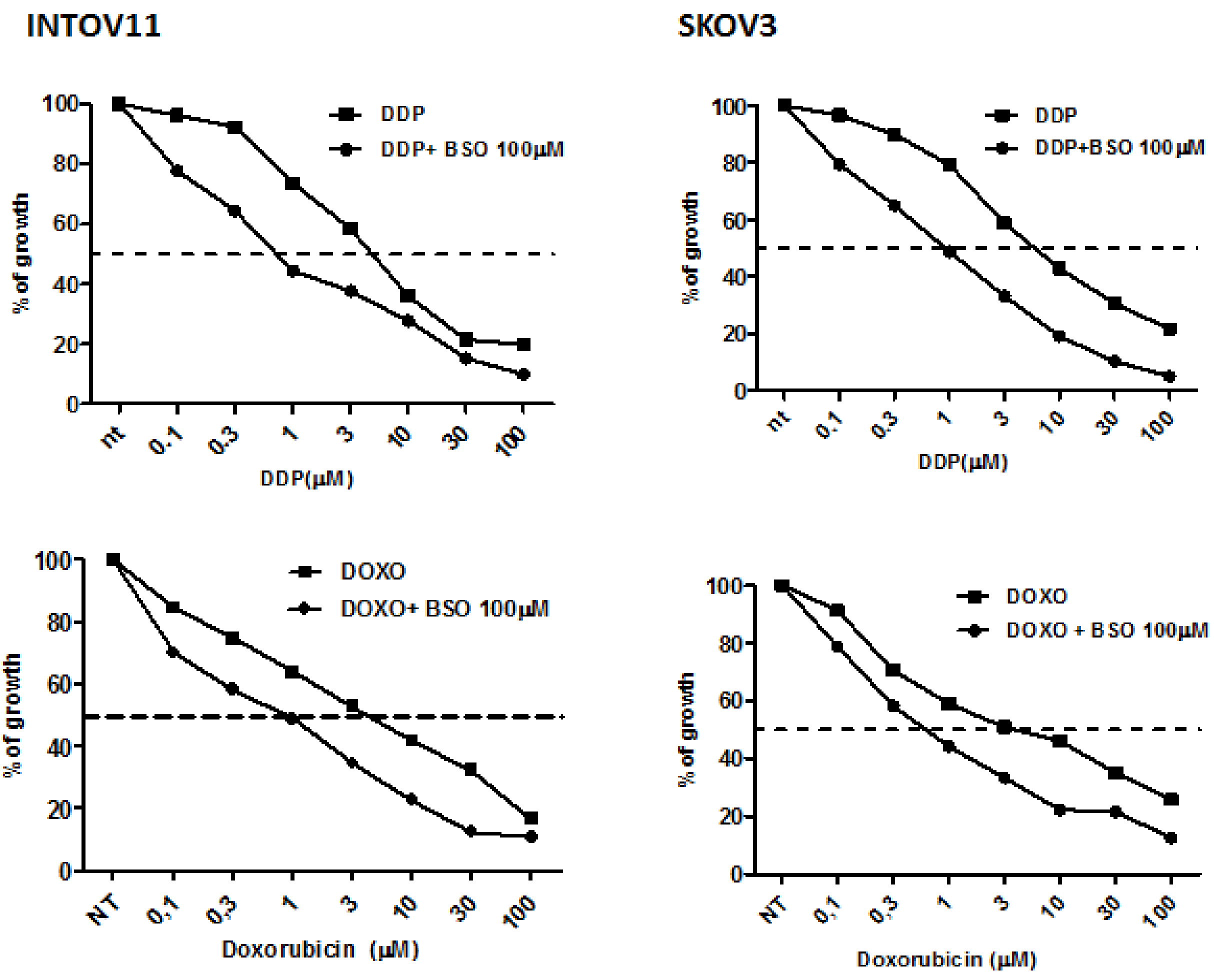

Supplementary Figure 5. Cell lines sensitization to drug treatment following GSH depletion. Sensitivity to DDP *(upper panels)* and doxorubicin (*lower panels*) in the presence or absence of 100 µM BSO in wild type INTOV11 and SKOV3 cells.
